# Positive allosteric modulation of the delta opioid receptor attenuates depression-like and pain-like behaviors but does not promote convulsions

**DOI:** 10.1016/j.jpet.2026.104942

**Published:** 2026-05-14

**Authors:** Emily M. Jutkiewicz, Benjamin A. Palleiko, Isaac J. Dripps, Kathryn E. Livingston, Bernard P. Roques, Benjamin M. Clements, John R. Traynor

**Affiliations:** 1 Department of Pharmacology, Edward F. Domino Research Center, University of Michigan, Ann Arbor, Michigan; 2 Unité de Technologies Chimiques et Biologiques pour la Santé, Université Paris Descartes, Paris, France; 3 Department of Medicinal Chemistry, University of Michigan, Ann Arbor, Michigan

**Keywords:** Allosteric modulation, Delta opioid receptor, Depression, Pain, Convulsions

## Abstract

The delta opioid receptor (DOR) has been a focus of research for the treatment of depression and certain pain disorders. Unfortunately, clinical translation of delta opioid agonists to humans has proved challenging partly because of their propensity to cause convulsions at higher doses. The discovery that the DOR can be allosterically modulated has opened the possibility of developing compounds that may be able to produce some of the positive therapeutic effects, either as standalone treatments to enhance the action of endogenous opioid peptides or as sparing agents allowing the use of lower doses of DOR agonists, which could reduce the chance of inducing convulsions. BMS-986187 is a positive allosteric modulator of DOR. The behavioral effects of BMS-986187 related to depression and pain have not been investigated. In this study, we examined the impact of BMS-986187 on DOR-mediated antidepressant-like effects in the forced swim test, antinociceptive and antiallodynic actions and propensity to cause convulsions. BMS-986187 (1 mg/kg) showed antidepressant-like effects when administered alone, but did not produce convulsions. BMS-986187 (10 mg/kg) also enhanced the antinociceptive and antiallodynic effects of the standard DOR agonist SNC80, with only a very minor effect on SNC80-mediated convulsions. The results suggest the development of positive modulators of DOR for the management of pain and/or depression.

**Significance Statement:**

The delta opioid receptor (DOR) has been suggested as a target to treat depression and pain. However, agonists at this receptor can cause convulsions. This study demonstrates that a positive allosteric modulator of DOR administered alone reduces depression-like behaviors and, in combination with a DOR agonist, shows robust pain-relieving actions, without convulsive effects. This suggests a novel and safe treatment for depression and/or pain.

## 1 Introduction

Delta opioid receptor (DOR) agonists produce robust antidepressant-like behaviors in several preclinical in vivo assays.^1^ In addition, agonists acting at DOR are antinociceptive in preclinical models of pain hypersensitivity after either chemical irritation^2^, ^3^, ^4^, ^5^ or inflammatory responses.^3^^,^^6^^,^^7^

DOR is an attractive target for the management of depression and/or pain because agonists at this receptor lack the unwanted effects that occur after administration of agonists acting at the mu opioid receptor (MOR), namely reward and respiratory depression. Unfortunately, many DOR agonists produce brief, nonfatal convulsions,^8^^,^^9^ and this behavior has contributed to their failure to reach the clinic.^10^ On the other hand, it has been shown that the antidepressant-like activity of DOR agonists can be separated from convulsive activity,^11^^,^^12^ and whether or not convulsions are seen can depend on the route and rate of administration.^13^ In addition, several DOR agonists that reportedly lack convulsive activity, but retain antidepressant-like effects and/or analgesic effects have been identified. These include KNT-127,^14^ JNJ-20788560,^15^ ADL-5859,^16^, ^17^, ^18^ ADL-5747,^16^^,^^18^^,^^19^ ARM390,^20^ the MOR antagonist DOR agonist diprenorphine,^21^ and several AZD compounds.^22^ Indeed, AZD-2327 has been evaluated in phase II clinical trials for anxious major depressive disorder but was not found to be significantly different from placebo.^22^ Similarly, ADL5859 and ADL5747 were evaluated for the treatment of various types of pain, including surgical pain, diabetes-induced peripheral neuropathy, osteoarthritis, and postherpetic neuralgia. In all conditions, the primary endpoint of pain reduction was not reached or the drugs were not effective enough to warrant continued development.^18^

As an alternative approach we examine the hypothesis that positive allosteric modulation of DOR will enhance the action of DOR agonists in vivo, including endogenous opioid peptides and small molecule DOR agonists such as SNC80, to provide effective antidepressant-like and antiallodynic activity without causing or enhancing convulsive activity. BMS-986187 is a xanthenedione-based positive allosteric modulator (PAM) of DOR (DOR PAM) with good selectivity over MOR in vitro.^23^^,^^24^ The compound has been shown to act via a DOR-mediated mechanism to reduce colonic motility in disease states without altering basal colonic motor complexes in nondiseased states.^25^ In addition, BMS-986187 increases the activity of endogenous opioid peptides in the basolateral amygdala, a region important for fear and anxiety.^26^ However, the impact that this modulator may have on DOR-mediated actions in mood or pain disorders, or indeed convulsions, is unknown. Here, we report an exploration of the action of the modulator BMS-986187 in depression-like and pain-like behaviors in the mouse, in either the absence or presence of the orthosteric DOR agonist SNC80. We demonstrate that BMS-986187 has antidepressant-like activity when administered alone, promotes the antinociceptive activity of SNC80, but shows no convulsive activity and has only a minor effect on the convulsive activity of the DOR agonist SNC80.

## 2 Materials and methods

### 2.1 Experimental design

The hypothesis before starting this research was that the DOR PAM BMS-986187 would enhance DOR agonist-induced antidepressant and antiallodynic effects without increasing convulsions. This hypothesis was proposed before the initiation of the studies based on previously reported results that PAMs at the MOR receptor show separation of pharmacological effects.^27^^,^^28^ The hypothesis was not adapted during or after data analysis. Sample sizes were defined to reduce use of animals while maintaining adequate power to test between groups.

### 2.2 Subjects

C57BL/6N mice were purchased from Envigo. DOR knockout mice (B6.129S2-*Oprd1*^*tm1Kff*^/J stock number 007557) and MOR knockout mice (B6.129S2-*Oprm1*^*tm1Kff*^*/*J stock number 007559) were purchased from The Jackson Laboratory and then bred in house. Wild type (+/+) littermates for both strains were obtained by breeding heterozygous (+/−) knockouts. Arrestin-2 homozygous knockout mice (Arrb^tm1jse^) were generously gifted by Dr Amynah A. Pradhan (Washington University). All mice used were 8 to 15 weeks of age, weighing 16–32 g at the time of use, and were used only once. Male and female mice were used in each study. Animals were group housed in cages of 2 to 4 and had free access to standard lab chow and water. The environment was temperature and humidity controlled and was on a 12-hour light/dark cycle with lights on at 7:00 am. All experiments complied with the *Guide for Care and Use of Laboratory Animals* by the National Institutes of Health and were approved by the *University of Michigan Institutional Committee on the Use and Care of Animals.* A total of 240 mice were used during the study. Equal numbers of male and female mice were used unless an experiment comprised an uneven number of subjects. No mice were excluded from the analyses.

### 2.3 Chemicals and drugs

All drugs were administered subcutaneously and at a volume of 10 mL/kg, except for nitroglycerin (NTG) and *β*-funaltrexamine (*β*-FNA), which were administered intraperitoneally at a volume of 10 mL/kg. Acetic acid was given intraperitoneally in a volume of 0.4 mL. BMS-986187 (3,4,5,6,7,9-hexahydro-3,3,6,6-tetramethyl-9-[4-[(2-methylphenyl)methoxy]phenyl]-1H-xanthene-1,8(2H)-dione, see Livingston et al^24^ for the chemical structure) was synthesized by the Valteich Medicinal Chemistry Core at the University of Michigan and dissolved in a 1:1:8:10 solution (DMSO, ethoxylated castor oil, 10% *β*-cyclodextrin solution, and sterile water). SNC80 (4-[(*R*)-[(2*S*,5*R*)-4-allyl-2,5-dimethylpiperazin-1-yl] (3-methoxyphenyl) methyl]-*N,N*-diethylbenzamide) was purchased from Cayman Chemical and first dissolved in 1M HCl, and then diluted to a concentration of 3% of acid with sterile water. Pentylenetetrazol (PTZ) (6,7,8,9-Tetrahydro-5H-tetrazolo(1,5-a)azepine) was purchased from Tocris Bioscience and dissolved in sterile water. Glacial acetic acid was diluted in sterile water to a concentration of 0.6% consistent with previous literature.^8^^,^^29^ NTG (propane-1,2,3-triyl trinitrate) was purchased from Sigma-Aldrich and dissolved in saline. The enkephalinase inhibitor RB101 (phenylmethyl (2S)-2-[(2-([(2S)-2-amino-4-methylsulfanylbutyl] disulfanylmethyl)-3-phenylpropanoyl)amino]-3-phenylpropanoate) was synthesized as previously described^30^ and dissolved in a 1:1:8 solution (ethanol, alkamuls, and sterile water). The irreversible MOR antagonist *β*-FNA was obtained through the National Institute on Drug Abuse Drug Supply Program and dissolved in sterile water.

### 2.4 GTPγ^35^S radioligand binding assay

#### 2.4.1 Preparation of mouse brain homogenates

Mice were euthanized by cervical dislocation and brain tissue, from the optic chiasmus forward, was collected. The tissue was immediately placed in ice-cold Tris base (50 mM, pH 7.4). Brain homogenates were prepared in a manner consistent with previous studies.^31^ After resuspension in Tris base (50 mM, pH 7.4), final membrane pellets were stored at −80 °C. The bicinchoninic acid assay was used to determine protein levels, using bovine serum albumin as a standard.

#### 2.4.2 Stimulation of GTPγ^35^S binding

Agonist stimulation of GTP*γ*^35^S was determined as previously described.^28^ Mouse brain homogenates (15–20 *μ*g/well) were incubated in buffer (50 mM Tris-HCl, 100 mM NaCl, 5 mM MgCl_2_, and pH 7.4) that contained 0.1 nM GTP*γ*^35^S, 100 *μ*M GDP, and 0.2 units/mL of adenosine deaminase. The buffer also contained either vehicle or 1 *μ*M BMS-986187 and SNC80 at multiple concentrations. The mixture was placed in a shaking water bath at 25 °C for 1 hour and the reaction was subsequently terminated using vacuum filtration through GF/C filters and a Brandel harvester and then washed 5 times with ice-cold buffer. After drying the filters and adding EcoLume scintillation cocktail, they were counted in a Wallac 1450 MicroBeta Liquid Scintillation and Luminescence Counter (Perkin Elmer). Extent of GTP*γ*^35^S binding was reported as percent maximum stimulation of DOR agonist SNC80 at 10 *μ*M. Assays were performed in triplicate and repeated 3 times.

### 2.5 Behavioral assays

#### 2.5.1 Forced swim test

The forced swim test was modified from Porsolt et al^32^ and performed as described in Dripps et al.^33^ Mice were placed in a 4 L beaker filled with water at 25 ± 1 °C to a depth of 15 cm. SNC80 was administered as a 60-minute pretreatment and BMS-986187 was administered as a 120-minute pretreatment, except when coadministered with SNC80 when it was given 90 minutes before the swim, or during for the time course evaluation. After the mice were placed in the beaker, they were allowed to swim for 6 minutes and experiments were recorded with a Sony HDR-CX220 video camera. Videos were scored after the experiment by an operator blind to experimental conditions. Scoring consisted of measuring immobility after the first 30 seconds of the swim. Small movements necessary for the mouse to stay afloat were scored as immobile.

#### 2.5.2 Acetic acid stretch assay

The previously described protocol for the acetic acid stretch assay was followed.^8^^,^^9^^,^^29^ Fifteen minutes after administration of SNC80 (1.0, 3.2, or 10 mg/kg, s.c.), BMS-986187 (10 mg/kg, s.c.) was given. Mice were then injected with 0.4 mL of 0.6% acetic acid 30 minutes after administration of SNC80. Abdominal stretches were counted for 30 minutes with observation starting 5 minutes after injection of acetic acid. Abdominal stretches were characterized by extension of hind limbs, forepaws, and contraction of the abdominal muscles.

#### 2.5.3 Nitroglycerin-induced hyperalgesia

The protocol for the nitroglycerin-induced hyperalgesia assay was performed as previously described in Dripps et al.^33^ The tails of mice were first submerged in water measuring 46 °C. After obtaining baseline values, NTG (10 mg/kg, i.p.) was administered to the animals. One hour later, the tail withdrawal latencies were measured again. Ninety minutes after administration of NTG, animals were injected with BMS-986187 (10 mg/kg, s.c.) or vehicle and SNC80 (1.0, 3.2, or 10 mg/kg, s.c.). Tail withdrawal latency was again measured 30 minutes after injection of BMS-986187 or SNC80.

#### 2.5.4 Carrageenan-induced tactile hypersensitivity

Tactile thresholds were tested using von Frey filaments. Filaments were placed against the underside of the left hind-paw until buckling, where they were held for 2–6 seconds or until a withdrawal was noted. Fifty percent withdrawal thresholds were determined by the up-down method.^34^ Carrageenan was then administered under isoflurane anesthesia into the plantar surface of the left hind-paw (20 *μ*L of 2.5% w/w carrageenan solution). After 24 hours, a robust tactile hypersensitivity was seen in the left hind-paw. Baseline injured thresholds were measured. Animals then received BMS-986187 (10 mg/kg, s.c.) or vehicle. Thresholds were determined 30 minutes later, and then animals received escalating cumulative doses of SNC80 every 30 minutes, with thresholds determined before each dosing.

#### 2.5.5 Measurement of convulsions

After receiving drug, mice were placed in individual cages for continuous observation. The bedding and enrichment were removed from the cage to facilitate observation of convulsive behavior. BMS-986187 was administered as a 30-minute pretreatment to PTZ and as a 60-minute pretreatment to SNC80. Convulsion severity was measured using a modified Racine Scale as described in Dripps et al^33^ with a score of zero meaning no convulsive behavior was observed and a score of 5 representing a clonic extension or tonic convulsion that lasted more than 3 seconds, as well as a loss of balance. Scores of 4 or 5 were recorded as convulsions to assess the percentage of mice convulsing.

### 2.6 Data and statistical analysis

All data analysis was performed using GraphPad Prism version 7.0 (GraphPad). The level of significance (*α*) was set to 0.05 for all tests and post hoc testing was performed using Tukey’s multiple comparisons test. Post hoc tests were only performed when *F* values produced *P* < .05. Groups were not powered to detect sex differences. No obvious differences were noted so both sexes were pooled for analysis.

## 3 Results

### 3.1 BMS-986187 enhances DOR activation in homogenates of mouse brain

**Fig. 1:**
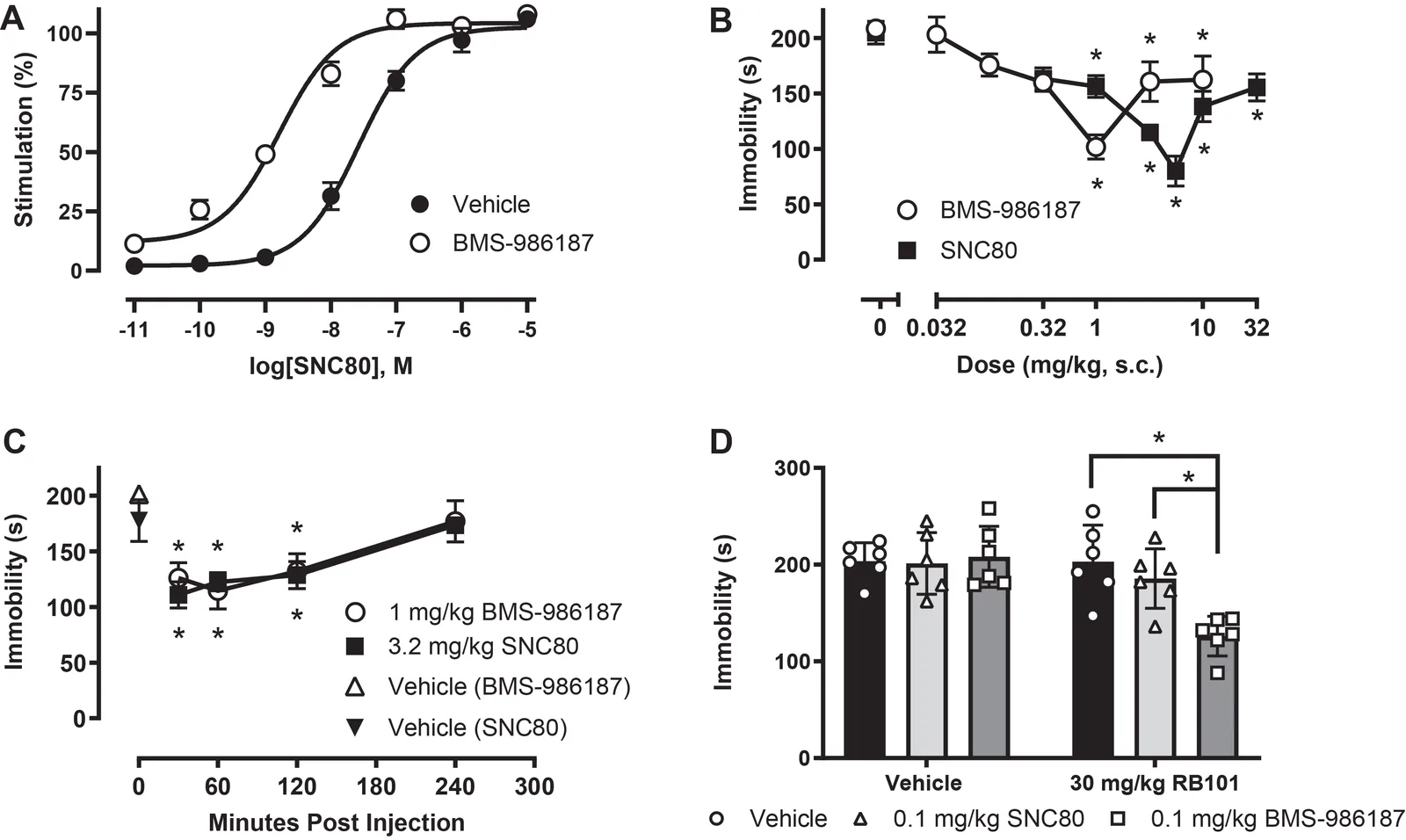
BMS-986187 enhances DOR agonists and induces antidepressant-like behaviors. (A) Binding of GTP*γ*^35^S in mouse brain homogenates with various doses of SNC80 in the presence of either vehicle or 1 *μ*M BMS-986187. The amount of GTP*γ*^35^S bound is defined as the percent stimulation of a maximal dose of a full agonist (10 *μ*M SNC80) (*n* = 3 separate experiments each in triplicate). (B) Effect of dose on immobility in the forced swim test for both BMS-986187 and SNC80 (*n* = 6–8). (C) Changes in immobility over time in the forced swim test of the first fully effective dose of BMS-986187 (1 mg/kg, s.c.) or SNC80 (3.2 mg/kg, s.c.) (*n* = 6–7). (D) Effect on immobility in the forced swim test of a subeffective dose of SNC80 (0.1 mg/kg, s.c.) or BMS-986187 (0.1 mg/kg, s.c.) in combination with the enkephalinase inhibitor RB101 (30 mg/kg, s.c.) (*n* = 6). ∗*P* < .05 represents comparison to vehicle (B and C) or ∗*P* < .05 represents comparison across treatment conditions alongside 30 mg/kg RB-101 (D). Significance determined by two-way ANOVA with post hoc Tukey’s multiple comparison test. Data are presented as mean ± SEM.

BMS-986187 has been shown to act as a DOR PAM in several cell systems heterologously expressing DOR.^23^^,^^24^ To confirm this PAM action in endogenous tissue we determined the ability of SNC80 to activate G*α* proteins as measured by the binding of the GTP analog GTP*γ*^35^S downstream of DOR in brain tissue from mice.^35^ SNC80 concentration-dependently increased the binding of GTP*γ*^35^S to mouse brain homogenates, giving an EC_50_ of 25.3 nM. In the presence of 1 *μ*M of BMS-986187 there was a 20-fold shift in potency giving an EC_50_ of 1.3 nM with no change in the maximal response (Fig. 1A).

### 3.2 Antidepressant-like effect of BMS-986187 as measured in the forced swim test

The forced swim test in rodents has been used to identify compounds with potential as antidepressant drugs. In this test animals develop a “learned helplessness,” that is, become immobile which is interpreted as a sign of despair or a depressive state. Compounds with antidepressant activity cause the animals to swim or climb to escape. DOR agonists acting at the orthosteric site such as SNC80 reduce immobility in the forced swim test in the rat^36^ and mouse^37^ and so have antidepressant-like activity.

Both SNC80 and BMS-986187 reduced immobility in the forced swim test in the mouse, which was reversed at higher doses producing a U-shaped dose-response relationship (Fig. 1B). There was a significant effect of the dose of BMS-986187 (*F*[6,37] = 6.707, *P* < .0001) and SNC80 (*F*[6,40] = 14.03, *P* < .0001). The maximum effect on immobility was achieved with 1 mg/kg BMS-986187 (*P* = .0001) and 5.6 mg/kg SNC80 (*P* = .0001). To evaluate the time course of BMS-986187 and SNC80, doses producing similar decreases in immobility were used (ie, 1 mg/kg BMS-986187 and 3.2 mg/kg SNC80). A two-way ANOVA revealed a significant effect of time on immobility (*F*[5,60] = 12.04, *P* < .0001) but no significant effects were observed with respect to the drug administered (BMS-986187 or SNC80). BMS-986187 and SNC80 both produced significant decreases in immobility at 30 minutes (*P* = .0218, *P* = .0012, respectively), 60 minutes (*P* = .0026, *P* = .0111), and 120 minutes (*P* = .0136, *P* = .0334), as compared with the control group (Fig. 1C).

### 3.3 Role of endogenous opioids in the antidepressant-like effects of BMS-986187 in the forced swim test

To test if BMS-986187 was causing its antidepressant-like effects by acting as a PAM to enhance the action of endogenous opioid peptides acting at DOR, we administered the compound as a pretreatment to the enkephalinase inhibitor RB101. RB101 acts to increase levels of endogenous enkephalins, which then act at the opioid receptors.^38^ A dose of 0.1 mg/kg BMS-986187 or SNC80 had no significant activity alone (Fig. 1D). However, when administered with 30 mg/kg RB101, an antidepressant-like effect was seen with BMS-986187 that was significantly different from vehicle (*P* = .0003). ANOVA revealed a significant main effect of RB101 treatment (*F*[1,30] = 11.15, *P* = .0023) and BMS-986187 or SNC80 treatment (*F*[2,30] = 4.838, *P* = .0151) on immobility scores, as well as a significant interaction (*F*[2,30] = 6.521, *P* = .0044) (Fig. 1D). Post hoc tests did not reveal any enhancement of RB101 in the presence of SNC80 (Fig. 1D), indicating an enhancement of endogenous opioid signaling with BMS-986187.

### 3.4 Role of DOR in the antidepressant-like effects of BMS-986187 in the forced swim test

**Fig. 2:**
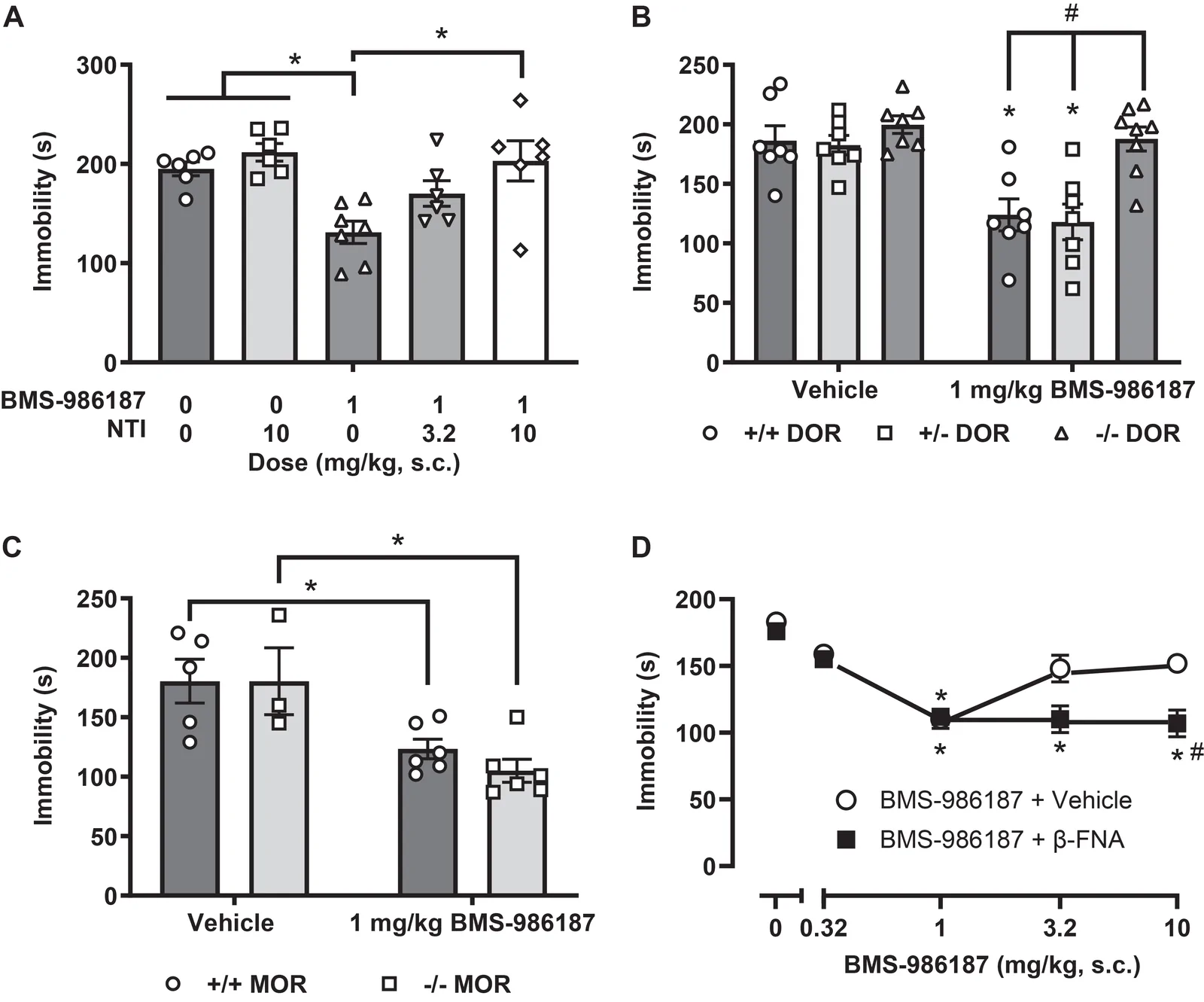
Antidepressant-like activity of BMS-986187 is DOR-dependent. (A) The effect of BMS-986187 in the forced swim test with or without pretreatment of the DOR-specific antagonist NTI (*n* = 6–7). (B) The effect of BMS-986187 in the forced swim test in DOR homozygous knockout, DOR heterozygous knockout, or wild type littermate mice (*n* = 7–8). (C) The effect of BMS-986187 (1 mg/kg, s.c.) in MOR knockout mice or wild type littermates (*n* = 3–6). (D) Changes in dose-response curve of BMS-986187 in the forced swim test after pretreatment with *β*-FNA (32 mg/kg, s.c.) or vehicle (sterile water) (*n* = 5). ∗*P* < .05, significantly different from the vehicle treated mice of the corresponding test doses. ^#^*P* < .05, significantly different from same treatment in alternate genotype or alternate drug treatment group. Significance determined by two-way ANOVA with post hoc Tukey’s multiple comparison test. Data are presented as mean ± SEM.

To confirm that BMS-986187 was producing its effect in the forced swim test through DOR, mice were treated with the selective DOR antagonist naltrindole (NTI). NTI dose-dependently attenuated the BMS-986187-induced decrease in immobility (Fig. 2A). ANOVA revealed significant differences in immobility scores between treatment groups (*F*[4,26] = 6.901, *P* = .0006). NTI pretreatment (10 mg/kg) significantly attenuated the reduction in immobility by 1 mg/kg BMS-986187 (*P* = .0033) (Fig. 2A). Similarly, in DOR knockout mice, administration of BMS-986187 did not decrease immobility (Fig. 2B), further confirming that DOR is necessary for BMS-986187 activity in the forced swim test. Moreover, BMS-986187 was fully effective in heterozygous DOR mice, therefore complete expression of DOR may not be required for this behavioral effect. There was significant effect of the administration of BMS-986187 (*F*[1,37] = 24.84, *P* < .0001) and genotype (*F*[2,37] = 8.921, *P* = .0007) on immobility scores, as well as a significant interaction between the dose of BMS-986187 and genotype (*F*[2,37] = 3.495, *P* = .0407) (Fig. 2B).

### 3.5 MOR is not involved in the antidepressant-like effects of BMS-986187 in the forced swim test

Although selective for DOR, BMS-986187 does act as a low potency PAM at MOR in vitro.^23^^,^^39^ To evaluate whether or not MOR played a role in the observed antidepressant-like effects in the forced swim test, wild type (+/+) littermates and homozygous (−/−) MOR knockout mice were administered 1 mg/kg BMS-986187. A significant drug effect with respect to BMS-986187 was found (*F*[1,16] = 19.89, *P* = .0004) in which both wild type (*P* = .0062) and MOR knockout mice (*P* = .0195) produced effects in the forced swim test when treated with 1 mg/kg BMS-986187 as compared with vehicle treated animals (Fig. 2C), suggesting no role for MOR.

However, MOR agonists are known to induce alterations in motor function and more complex changes in mood. We therefore hypothesized that MOR activation may be partially responsible for the U-shaped dose-response curve at higher doses of BMS-986187 (Fig. 1A). To test for this, mice were pretreated with the irreversible MOR antagonist *β*-FNA (32 mg/kg, s.c.) 48 hours before receiving 10 mg/kg BMS-986187. This pretreatment attenuated the ascending limb of the dose-response curve that occurred at doses of 3.2 and 10 mg/kg BMS-986187 (Fig. 2D). Two-way ANOVA demonstrated a significant interaction between *β*-FNA and BMS-986187 (*F*[4,51] = 3.264, *P* = .0185), as well as a significant effect of BMS-986187 (*F*[4,51] = 19.29, *P* < .0001) and *β*-FNA (*F*[1,51] = 10.8, *P* = .0018). Post hoc analysis revealed that both the vehicle and 32 mg/kg *β*-FNA pretreated groups showed decreased immobility after 1 mg/kg BMS-986187 (*P* < .0001). At doses of 3.2 and 10 mg/kg BMS-986187, 32 mg/kg *β*-FNA treated animals continued to show decreased immobility (*P* = .0002, *P* < .0001, respectively). Animals treated with the vehicle and 10 mg/kg BMS-986187 differed significantly from those treated with 32 mg/kg *β*-FNA and 10 mg/kg BMS-986187 (*P* = .0073).

### 3.6 Effect of BMS-986187 on nociceptive behaviors

In these assays a single dose of 10 mg/kg BMS-986187 was used. Higher doses could not be administered because of the very low solubility of the molecule.

#### 3.6.1 Acetic acid stretch assay

**Fig. 3:**
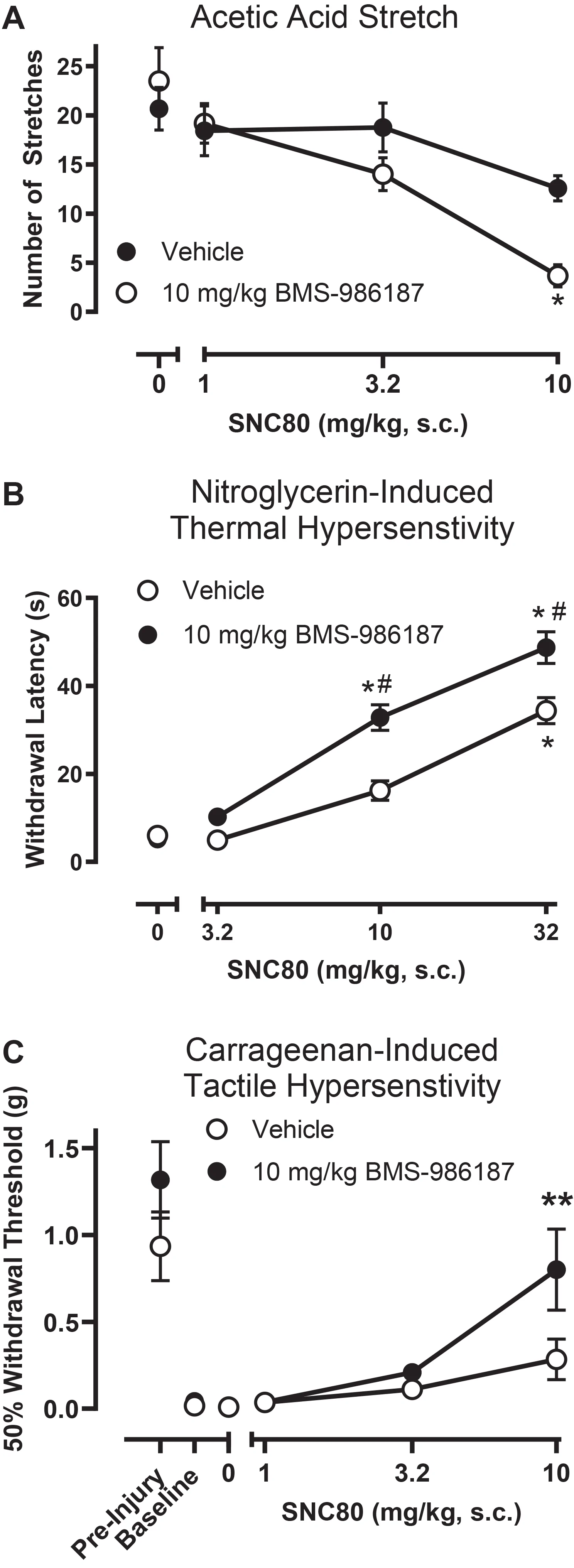
Effect of BMS-986187 on antinociceptive and antihyperalgesic effects of DOR agonists. (A) The number of stretches over a period of 30 minutes after administration of acetic acid (0.4 mL of 0.6%, i.p.) were recorded 30-minutes after pretreatment with SNC80 or vehicle with a 15-minute pretreatment with BMS-986187 or vehicle (*n* = 6–7). (B) Thermal withdrawal latencies in the warm-water tail withdrawal assay at 46 °C, after administration of NTG to induce thermal hyperalgesia. Ninety minutes after NTG, BMS-986187 and/or SNC80 were administered and changes in thermal withdrawal latency were recorded (*n* = 6–7). (C) Fifty percent paw withdrawal threshold determined using the von Frey assay after induction of hypersensitivity by 2.5% carrageenan administration (intra plantar) 24 hours before testing. Preinjury thresholds were determined in mice before treatment with carrageenan or administration of BMS-986187, SNC80 or vehicle. Baseline thresholds were determined 24 hours after carrageenan treatment and before any drug treatment. Mice were then treated with BMS-986187 or its vehicle and 30 minutes later thresholds were determined in the absence of SNC80 (0) and then in the presence of ascending doses of SNC80 given every 30 minutes (*n* = 5–6). ∗*P* < .05, significantly different from the vehicle treated mice of the same test condition; ^#^*P* < .05, significantly different from mice in the alternate treatment group. Significance was tested using two-way ANOVA with post hoc Tukey’s multiple comparison test. Data are presented as mean ± SEM.

The acetic acid stretch assay is a model of chemical irritation-mediated pain.^9^ BMS-986187 at 10 mg/kg did not produce a decrease in the number of abdominal stretches in mice. On the other hand, BMS-986187 significantly enhanced the ability of SNC80 to decrease stretching (*P* < .0001) (Fig. 3A). Two-way ANOVA revealed a significant main effect of the dose of SNC80 on the number of abdominal stretches (*F*[3,45] = 13.89, *P* < .0001).

#### 3.6.2 Nitroglycerin-induced hyperalgesia

NTG administration generates robust thermal hyperalgesia. Consequently, after NTG treatment the normally non-noxious temperature of 46 °C in the warm-water tail withdrawal assay causes the mice to withdrawal their tails after approximately 5 seconds. To assess the extent of antihyperalgesia we used a cut-off of 60 seconds. The NTG-induced hyperalgesia response was inhibited by SNC80.^40^ BMS-986187 (10 mg/kg) alone did not reduce the hyperalgesia, but when given with SNC80 induced an approximately 3-fold leftward shift in the potency of SNC80 (Fig. 3B). Two-way ANOVA revealed a significant effect of the dose of SNC80 (*F*[3,42] = 106.9, *P* < .0001) and the dose of BMS-986187 (*F*[1,42] = 30.34, *P* < .0001) as well as a significant interaction between SNC80 and BMS-986187 (*F*[3,42] = 6.305, *P* = .0012). Post hoc analysis revealed significant differences in withdrawal latencies between the vehicle and groups administered 10 mg/kg SNC80 (*P* = .0001) or 32 mg/kg SNC80 (*P* = .0018).

#### 3.6.3 Carrageenan-induced inflammatory pain

Carrageenan injection in the hind-paw induced a tactile hypersensitivity 24 hours later that was quantified with von Frey filaments. BMS-986187 alone did not reduce this carrageenan-induced hypersensitivity, nor did low doses of SNC80 (Fig. 3C). However, there was a significant increase in the reversal of hypersensitivity at the highest dose of SNC80 in the presence of BMS-986187 (*P* = .0042). Two-way ANOVA indicated a significant effect of the dose of SNC80 (*F*[5,108] = 60.51, *P* < .0001) and the pretreatment of BMS-986187 (*F*[3,108] = 6.933, *P* = .0003) as well as a significant interaction between SNC80 and BMS-986187 (*F*[15,108] = 2.464, *P* = .0038).

### 3.7 Effect of BMS-986187 on convulsive behavior

**Fig. 4:**
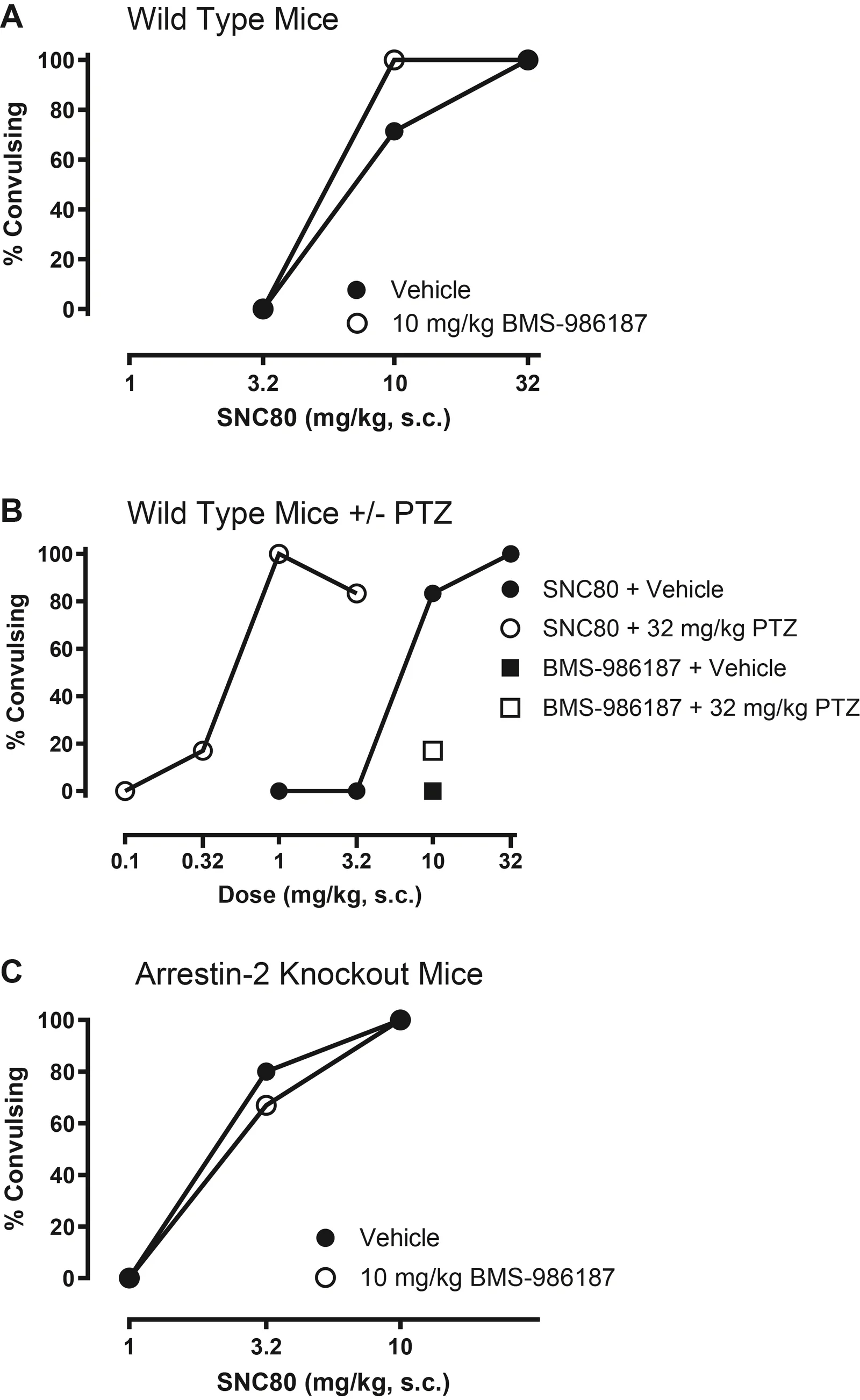
Effect of BMS-986187 on convulsion behaviors. (A) Percent of mice convulsing after administration of either vehicle or 10 mg/kg BMS-986187 as a pretreatment to SNC80 (*n* = 7). (B) Percent of mice convulsing after administration of SNC80 or BMS-986187 as a pretreatment to PTZ. Increasing doses of SNC80 are represented on the *X*-axis; only a single dose of BMS-986187 (10 mg/kg) was examined (*n* = 6). (C) Percent of arrestin-2 knockout mice showing convulsions after either vehicle or 10 mg/kg BMS-986187 pretreatment to SNC80 (*n* = 6).

BMS-986187 did not produce convulsions at 10 mg/kg, a tenfold higher dose than the maximum effective dose in the forced swim test (1 mg/kg) and the effective dose in antinociceptive tests (Fig. 4B). In contrast, SNC80 produced dose-dependent increases in the percentage of mice convulsing, with 100% of mice convulsing at 32 mg/kg (Fig. 4A). BMS-986187 at 10 mg/kg increased the number of mice convulsing at the 10 mg/kg dose of SNC80 (from 6/7 to 7/7 mice) but had no effect at the lower 3.2 mg/kg dose (Fig. 4A). To further examine the impact of BMS-986187 on convulsive behavior, the effect of the convulsant PTZ on the ability of BMS-986187 and SNC80 to cause convulsions was tested. At 32 mg/kg, PTZ alone failed to reliably produce convulsions in mice, and in the presence of 10 mg/kg BMS-986187 only 1 out of 7 mice experienced convulsions (Fig. 4B). In contrast, the potency of SNC80 shifted 30-fold in combination with 32 mg/kg PTZ such that a dose of 1 mg/kg elicited convulsive behaviors in greater than 80% of mice (Fig. 4B).

Arrestin-2 knockout mice, have previously been demonstrated to show increased susceptibility to SNC80-induced convulsions.^33^ These mice did not show convulsions after administration of BMS-986187 at 10 mg/kg (Fig. 4C). BMS-986187 also did not potentiate SNC80 induced convulsions in the arrestin-2 knockout mice (Fig. 4C).

## 4 Discussion

In the present work we demonstrate that the DOR PAM, BMS-986187, acts to provide an antidepressant-like effect in the forced swim test by enhancing the action of endogenous opioid peptides at the receptor. The magnitude of this effect was similar to that of the small molecule DOR agonist SNC80. The BMS-986187-mediated antidepressant-like effect was absent in DOR knockout mice and blocked by NTI confirming that the actions of BMS-986187 were mediated by DOR. In contrast, BMS-986187 alone had no effect in reversing nociceptive responses in the acetic acid stretch assays or hyperalgesia caused by carrageenan or nitroglycerin. Although, BMS-986187 did promote the potency of the DOR agonist SNC80 in these assays, no convulsions were observed after administration of BMS-986187 alone, but 1 of 7 mice in the study did convulse when the PAM was administered with a subeffective dose of the proconvulsant PTZ. In contrast, PTZ increased the potency of SNC80 to cause convulsions by 30-fold. BMS-986187 had a minor effect on the convulsant activity of SNC80, which was only apparent at an intermediate dose of SNC80. Overall, the findings provide evidence that BMS-986187 could act as a lead for the development of safer medications targeting DOR.

BMS-986187 does show direct agonist action at DOR via an allosteric site, so called ago-PAM activity in vitro,^39^^,^^41^ which could explain its direct DOR-mediated antidepressant-like effect in the absence of any exogenous orthosteric DOR agonist. However, ago-PAM activity is seen in overexpressed cells and is not manifest, or is extremely weak, in higher efficacy requiring assays.^23^^,^^24^^,^^41^ Moreover, BMS-987187 acts at an allosteric site on DOR, not the orthosteric site and so is only partially displaced by NTI,^41^ whereas NTI fully reverses the antidepressant-like actions of BMS-986187. Thus, direct ago-PAM activity is unlikely responsible for the actions of BMS-986187 in vivo. On the other hand, BMS-986187 promotes the actions of endogenous opioid peptides in cell systems expressing DOR^23^^,^^24^ and in neurons of the basolateral amygdala.^26^ In the forced swim test, we observed a synergy between BMS-986187 and the enkephalinase inhibitor RB101, a compound that increases the lifetime, and therefore concentrations of endogenous opioid peptides. The forced swim test is a known stressor in rodents and leads to the release of adrenocorticotropin and corticosterone^42^ as well as opioid peptides,^27^^,^^43^, ^44^, ^45^, ^46^ the action of which will be enhanced by BMS-986187. Therefore, the data point to the antidepressant-like action of BMS-986187 being due to a DOR PAM action that enhances the activity of endogenous opioid peptides released during the behavioral protocol, leading to decreased immobility.

We observed a biphasic dose-response for BMS-986187 in the forced swim test, with a reduction in effect at higher doses that was blocked by the irreversible MOR antagonist *β*-FNA. This agrees with findings that mice lacking MOR show decreased depression-like behaviors^47^ and so can be explained by the weak activity of BMS-986187 as MOR-PAM,^24^ thereby promoting the action of released opioid peptides at MOR. The biphasic response seen after SNC80 administration is not likely to be due to activity at MOR given its almost 500-fold selectivity for DOR over MOR,^48^ but does occur at proconvulsive doses of the drug and so this could be an explanation.

In contrast to its effects in the forced swim test, BMS-986187 administered alone to mice did not produce an antinociceptive response. However, BMS-9861987 enhanced the action of SNC80 in the acetic acid stretch, NTG-induced hyperalgesia, and carrageenan-induced tactile allodynia assays. Although opioid peptides are also likely to be released during the pain assays,^27^^,^^49^, ^50^, ^51^ the efficacy requirements for activity in the forced swim test are less stringent than the nociceptive assays. This is supported by the different dosing requirements of the assays, with lower doses of SNC80 required to elicit antidepressant-like behaviors compared with antinociceptive and antiallodynic behaviors.^13^^,^^40^^,^^52^ Moreover, a full antidepressant-like effect was seen in genetically modified mice lacking 50% of their DORs, providing further evidence for the low efficacy requirements for reducing immobility in the forced swim test. The dose of BMS-986187 to enhance antinociceptive responses may be high enough to act at MOR.^28^ However given that the modulator has no effect in the present experiments in the absence of SNC80, an action at MOR is unlikely.

Administration of 10 mg/kg BMS-986187, a dose 10 times that required for antidepressant-like effects in the forced swim test, did not produce convulsions alone. Because of solubility issues, we were unable to examine higher doses of BMS-986187 to determine the extent of the separation in dosage between the different behaviors. In contrast, convulsions were seen with SNC80 at 10 mg/kg, which is only 3 times the dose required to produce similar decreases in immobility in the forced swim test. However, the fact that BMS-986187 was observed to afford a convulsive response in only 1 of 7 PTZ-treated mice, nor show activity in the arrestin-2 knockout mice does indicate that DOR PAMs may not be convulsant. The reasons why convulsions are not seen after BMS-986187 alone could be because of a lack of endogenous opioid peptides released during the assay. Alternatively, BMS-986187 has PAM activity at the kappa opioid receptor (KOR) with similar potency as observed at DOR.^24^ Small molecule KOR agonists are anticonvulsant^53^^,^^54^ and dynorphin is considered as an endogenous anticonvulsant.^55^, ^56^, ^57^ Therefore, if BMS-986187 does promote the action of endogenous dynorphin peptides at KOR this could conceal the potential proconvulsant action of BMS-986187. However, promotion of dynorphin activity would also be expected to produce a decreased motility in the forced swim test^58^ and so is an unlikely mechanism.

It is feasible that BMS-986187 produces its effects in the forced swim test and nociceptive assays independent of the signaling pathways that are involved in DOR-mediated convulsions. For example, BMS-986187 has been proposed to bias signaling through the G-protein coupled pathway over pathways downstream of *β*-arrestin^41^ and studies with mice expressing 50% G_*α*o_ have implicated G_*α*o_ pathways in producing DOR-mediated antidepressant-like effects, and pain-relieving effects in the acetic acid stretch and NTG-induced hyperalgesia assays.^33^^,^^40^ In contrast, there is evidence that SNC80 recruits *β*-arrestin more efficiently than DOR agonists that do not cause convulsions,^59^ suggesting pathways downstream of *β*-arrestin may be responsible for the proconvulsive actions. Whatever the reason the observed selectivity of DOR PAMs to enhance certain physiological responses over others has also been seen with PAMs acting at MOR, suggesting that, by whatever means, PAMs of opioid receptors appear able distinguish between on-target effects.

There are several limitations to the current study. First, we only used the forced swim test to determine the antidepressant-like action of BMS-986187. The development of antidepressants using animal models has been fraught with failures so translation of DOR PAMs to the clinic will require preclinical proof of concept using a battery of tests, including novelty suppressed feeding behavior and resident intruder assays.^60^ Similarly, if DOR PAMs are to be developed for the management of pain, measures beyond acute nociception and carrageenan-induced inflammatory pain are required, for example, migraine pain responds to DOR agonists in preclinical models.^51^ Second, the study was not powered to detect sex differences. There are some indications in rodent models that DOR agonists may show sexual dimorphism in measures of antinociception^61^ and there are reported differences between males and female rodents in the concentrations of opioid peptides and DOR receptor expression in some brain regions.^62^ Thus, future studies would need to address this with larger group sizes. Third, we only studied BMS-986187. Confirming our findings with other DOR PAMs would strengthen the argument for developing this class of compounds. Alternative DOR PAMs are becoming available^60^, ^61^, ^62^, ^63^, ^64^ but these are based on BMS-986187 and are not yet well characterized. In addition, structural data suggest the newer compounds bind to the same site and work by the same mechanism as BMS-986187.^60^ Unfortunately, BMS-986187 is not a drug-like molecule. It is a high molecular weight, highly lipophilic compound with low solubility requiring complex formulations, thus preventing administration of doses above 10 mg/kg. The compound also has PAM activity at MOR and KOR.^24^ Nonetheless, the fact that structures of a BMS-986187 and similar compounds bound to DOR are now available^63^^,^^64^, ^65^, ^66^ raises the possibility that druggable, highly selective DOR PAMs, may be developed as safe, rapid, and effective novel antidepressants and/or antihyperalgesic agents.

## 5 Conclusion

We have shown that BMS-986187 produces antidepressant-like effects in the forced swim test in mice without producing convulsions. Moreover, BMS-986187 enhances the antinociceptive, antihyperalgesic and antidepressant-like effects of the orthosteric DOR agonist SNC80 without markedly enhancing the ability of SNC80 to cause convulsions. Although we have not been able to show the extent of this dose separation between these behaviors, we would envisage this would be an even greater margin of safety with DOR agonists that do not show overt proconvulsive-like effects. For example, at least one of these (AZD2327) was evaluated in phase II clinical trials for antidepressant and anxiolytic activity,^22^ but the effects were not significantly different from placebo.

## Conflict of interest

John R. Traynor holds a National Institutes of Health, Small Business Technology Transfer (STTR) grant with Eleven Therapeutics Corporation. All other authors declare no conflicts of interest.

## References

[1] Jutkiewicz E.M. (2006). The antidepressant -like effects of delta-opioid receptor agonists. Mol Interv.

[2] Sora I., Li X.F., Funada M., Kinsey S., Uhl G.R. (1999). Visceral chemical nociception in mice lacking mu-opioid receptors: effects of morphine, SNC80 and U-50,488. Eur J Pharmacol.

[3] Gallantine E.L., Meert T.F. (2005). A comparison of the antinociceptive and adverse effects of the mu-opioid agonist morphine and the delta-opioid agonist SNC80. Basic Clin Pharmacol Toxicol.

[4] Brandt M.R., Furness M.S., Mello N.K., Rice K.C., Negus S.S. (2001). Antinociceptive effects of delta-opioid agonists in Rhesus monkeys: effects on chemically induced thermal hypersensitivity. J Pharmacol Exp Ther.

[5] Negus S.S., Rosenberg M.B., Altarifi A.A., O’Connell R.H., Folk J.E., Rice K.C. (2012). Effects of the δ opioid receptor agonist SNC80 on pain-related depression of intracranial self-stimulation (ICSS) in rats. J Pain.

[6] Gavériaux-Ruff C., Karchewski L.A., Hever X., Matifas A., Kieffer B.L. (2008). Inflammatory pain is enhanced in delta opioid receptor-knockout mice. Eur J Neurosci.

[7] Fraser G.L., Gaudreau G.A., Clarke P.B., Ménard D.P., Perkins M.N. (2000). Antihyperalgesic effects of delta opioid agonists in a rat model of chronic inflammation. Br J Pharmacol.

[8] Broom D.C., Nitsche J.F., Pintar J.E., Rice K.C., Woods J.H., Traynor J.R. (2002). Comparison of receptor mechanisms and efficacy requirements for delta-agonist-induced convulsive activity and antinociception in mice. J Pharmacol Exp Ther.

[9] Hong E.J., Rice K.C., Calderon S., Woods J.H., Traynor J.R. (1998). Convulsive behavior of nonpeptide δ-opioid ligands: comparison of SNC80 and BW373U86 in mice. Analgesia.

[10] Comer S.D., Hoenicke E.M., Sable A.I. (1993). Convulsive effects of systemic administration of the delta opioid agonist BW373U86 in mice. J Pharmacol Exp Ther.

[11] Broom D.C., Jutkiewicz E.M., Folk J.E., Traynor J.R., Rice K.C., Woods J.H. (2002). Convulsant activity of a non-peptidic delta-opioid receptor agonist is not required for its antidepressant-like effects in Sprague-Dawley rats. Psychopharmacology.

[12] Jutkiewicz E.M., Baladi M.G., Folk J.E., Rice K.C., Woods J.H. (2006). The convulsive and electroencephalographic changes produced by nonpeptidic delta-opioid agonists in rats: comparison with pentylenetetrazol. J Pharmacol Exp Ther.

[13] Jutkiewicz E.M., Rice K.C., Traynor J.R., Woods J.H. (2005). Separation of the convulsions and antidepressant-like effects produced by the delta-opioid agonist SNC80 in rats. Psychopharmacology (Berl).

[14] Saitoh A., Sugiyama A., Nemoto T. (2011). The novel δ opioid receptor agonist KNT-127 produces antidepressant-like and antinociceptive effects in mice without producing convulsions. Behav Brain Res.

[15] Codd E.E., Carson J.R., Colburn R.W. (2009). JNJ-20788560 [9-(8-azabicyclo[3.2.1]oct-3-ylidene)-9H-xanthene-3-carboxylic acid diethylamide], a selective delta opioid receptor agonist, is a potent and efficacious antihyperalgesic agent that does not produce respiratory depression, pharmacologic tolerance, or physical dependence. J Pharmacol Exp Ther.

[16] Nozaki C., Le Bourdonnec B., Reiss D. (2012). δ-Opioid mechanisms for ADL5747 and ADL5859 effects in mice: analgesia, locomotion, and receptor internalization. J Pharmacol Exp Ther.

[17] Le Bourdonnec B., Windh R.T., Ajello C.W. (2008). Potent, orally bioavailable delta opioid receptor agonists for the treatment of pain: discovery of N,N-diethyl-4-(5-hydroxyspiro[chromene-2,4’-piperidine]-4-yl)benzamide (ADL5859). J Med Chem.

[18] Spahn V., Stein C. (2017). Targeting delta opioid receptors for pain treatment: drugs in phase I and II clinical development. Expert Opin Investig Drugs.

[19] Le Bourdonnec B., Windh R.T., Leister L.K. (2009). Spirocyclic delta opioid receptor agonists for the treatment of pain: discovery of N,N-diethyl-3-hydroxy-4-(spiro[chromene-2,4’-piperidine]-4-yl) benzamide (ADL5747). J Med Chem.

[20] Pradhan A.A., Walwyn W., Nozaki C. (2010). Ligand-directed trafficking of the δ-opioid receptor in vivo: two paths toward analgesic tolerance. J Neurosci.

[21] Olson K.M., Hillhouse T.M., Burgess G.E. (2023). Delta opioid receptor-mediated antidepressant-like effects of diprenorphine in mice. J Pharmacol Exp Ther.

[22] Richards E.M., Mathews D.C., Luckenbaugh D.A. (2016). A randomized, placebo-controlled pilot trial of the delta opioid receptor agonist AZD2327 in anxious depression. Psychopharmacology.

[23] Burford N.T., Livingston K.E., Canals M. (2015). Discovery, synthesis, and molecular pharmacology of selective positive allosteric modulators of the δ-opioid receptor. J Med Chem.

[24] Livingston K.E., Stanczyk M.A., Burford N.T., Alt A., Canals M., Traynor J.R. (2018). Pharmacologic evidence for a putative conserved allosteric site on opioid receptors. Mol Pharmacol.

[25] DiCello J.J., Carbone S.E., Saito A. (2022). Positive allosteric modulation of endogenous delta opioid receptor signaling in the enteric nervous system is a potential treatment for gastrointestinal motility disorders. Am J Physiol Gastrointest Liver Physiol.

[26] Winters B.L., Gregoriou G.C., Kissiwaa S.A. (2017). Endogenous opioids regulate moment-to-moment neuronal communication and excitability. Nat Commun.

[27] Kandasamy R., Hillhouse T.M., Livingston K.E. (2021). Positive allosteric modulation of the mu-opioid receptor produces analgesia with reduced side effects. Proc Natl Acad Sci U S A.

[28] Kochan K.E., Clements B.M., Prince T.D. (2026). A mu-opioid receptor positive allosteric modulator provides opioid-sparing antinociception without enhancing opioid side effects. J Pharmacol Exp Ther.

[29] Broom D.C., Guo L., Coop A. (2000). BU48: a novel buprenorphine analog that exhibits delta-opioid-mediated convulsions but not delta-opioid-mediated antinociception in mice. J Pharmacol Exp Ther.

[30] Fournie-Zaluski M.C., Coric P., Turcaud S. (1992). “Mixed inhibitor-prodrug” as a new approach toward systemically active inhibitors of enkephalin-degrading enzymes. J Med Chem.

[31] Lester P.A., Traynor J.R. (2006). Comparison of the in vitro efficacy of mu, delta, kappa and ORL1 receptor agonists and non-selective opioid agonists in dog brain membranes. Brain Res.

[32] Porsolt R.D., Bertin A., Jalfre M. (1977). Behavioral despair in mice: a primary screening test for antidepressants. Arch Int Pharmacodyn Ther.

[33] Dripps I.J., Boyer B.T., Neubig R.R., Rice K.C., Traynor J.R., Jutkiewicz E.M. (2018). Role of signalling molecules in behaviours mediated by the δ opioid receptor agonist SNC80. Br J Pharmacol.

[34] Chaplan S.R., Bach F.W., Pogrel J.W., Chung J.M., Yaksh T.L. (1994). Quantitative assessment of tactile allodynia in the rat paw. J Neurosci Methods.

[35] Traynor J.R., Nahorski S.R. (1995). Modulation by mu-opioid agonists of guanosine-5′-O-(3-[35S]thio)triphosphate binding to membranes from human neuroblastoma SH-SY5Y cells. Mol Pharmacol.

[36] Broom D.C., Jutkiewicz E.M., Folk J.E., Traynor J.R., Rice K.C., Woods J.H. (2002). Nonpeptidic δ-opioid receptor agonists reduce immobility in the forced swim assay in rats. Neuropsychopharmacology.

[37] Saitoh A., Kimura Y., Suzuki T., Kawai K., Nagase H., Kamei J. (2004). Potential anxiolytic and antidepressant-like activities of SNC80, a selective delta-opioid agonist, in behavioral models in rodents. J Pharmacol Sci.

[38] Jutkiewicz E.M. (2007). RB101-mediated protection of endogenous opioids: potential therapeutic utility?. CNS Drug Rev.

[39] Li M., Gan X., Liu K. (2025). Structure-activity relationships and molecular pharmacology of positive allosteric modulators of the mu-opioid receptor. ACS Chem Neurosci.

[40] Dripps I.J., Wang Q., Neubig R.R., Rice K.C., Traynor J.R., Jutkiewicz E.M. (2017). The role of regulator of G protein signaling 4 in delta-opioid receptor-mediated behaviors. Psychopharmacology (Berl).

[41] Stanczyk M.A., Livingston K.E., Chang L., Weinberg Z.Y., Puthenveedu M.A., Traynor J.R. (2019). The δ-opioid receptor positive allosteric modulator BMS 986187 is a G-protein-biased allosteric agonist. Br J Pharmacol.

[42] Rittenhouse P.A., López-Rubalcava C., Stanwood G.D., Lucki I. (2002). Amplified behavioral and endocrine responses to forced swim stress in the Wistar-Kyoto rat. Psychoneuroendocrinology.

[43] Parikh D., Hamid A., Friedman T.C. (2011). Stress-induced analgesia and endogenous opioid peptides: the importance of stress duration. Eur J Pharmacol.

[44] Amir S. (1982). Involvement of endogenous opioids with forced swimming-induced immobility in mice. Physiol Behav.

[45] Bruchas M.R., Xu M., Chavkin C. (2008). Repeated swim stress induces kappa opioid-mediated activation of extracellular signal-regulated kinase 1/2. Neuroreport.

[46] Contet C., Gavériaux-Ruff C., Matifas A., Caradec C., Champy M.F., Kieffer B.L. (2006). Dissociation of analgesic and hormonal responses to forced swim stress using opioid receptor knockout mice. Neuropsychopharmacology.

[47] Lutz P.E., Kieffer B.L. (2013). Opioid receptors: distinct roles in mood disorders. Trends Neurosci.

[48] Bilsky E.J., Calderon S.N., Wang T. (1995). SNC 80, a selective, nonpeptidic and systemically active opioid delta agonist. J Pharmacol Exp Ther.

[49] Jiang Y.L., He X.F., Shen Y.F. (2015). Analgesic roles of peripheral intrinsic met-enkephalin and dynorphin A in long-lasting inflammatory pain induced by complete Freund’s adjuvant in rats. Exp Ther Med.

[50] Cesselin F., Le Bars D., Bourgoin S. (1985). Spontaneous and evoked release of methionine-enkephalin-like material from the rat spinal cord in vivo. Brain Res.

[51] Mohamed F.A., Smrcka A.V., Jutkiewicz E.M. (2025). Biasing G protein *β**γ* subunit downstream signaling enhances the analgesic effects of endogenous opioid receptor agonists during nitroglycerin-induced thermal hypersensitivity. Mol Pharmacol.

[52] Pradhan A.A., Smith M.L., Zyuzin J., Charles A. (2014). δ-Opioid receptor agonists inhibit migraine-related hyperalgesia, aversive state and cortical spreading depression in mice. Br J Pharmacol.

[53] Tortella F.C., Robles L., Holaday J.W. (1986). U50,488, a highly selective kappa opioid: anticonvulsant profile in rats. J Pharmacol Exp Ther.

[54] Kumar H., Katyal J., Kumar Gupta Y. (2023). Effect of U50488, a selective kappa opioid receptor agonist and levetiracetam against lithium-pilocarpine-induced status epilepticus, spontaneous convulsive seizures and related cognitive impairment. Neurosci Lett.

[55] Pirker S., Gasser E., Czech T. (2009). Dynamic up-regulation of prodynorphin transcription in temporal lobe epilepsy. Hippocampus.

[56] Loacker S., Sayyah M., Wittmann W., Herzog H., Schwarzer C. (2007). Endogenous dynorphin in epileptogenesis and epilepsy: anticonvulsant net effect via kappa opioid receptors. Brain.

[57] Schwarzer C. (2009). 30 Years of dynorphins–new insights on their functions in neuropsychiatric diseases. Pharmacol Ther.

[58] McLaughlin J.P., Marton-Popovici M., Chavkin C. (2003). Kappa opioid receptor antagonism and prodynorphin gene disruption block stress-induced behavioral responses. J Neurosci.

[59] Blaine A.T., Miao Y., Yuan J. (2022). Exploration of beta-arrestin isoform signaling pathways in delta opioid receptor agonist-induced convulsions. Front Pharmacol.

[60] Belzung C. (2014). Innovative drugs to treat depression: did animal models fail to be predictive or did clinical trials fail to detect effects?. Neuropsychopharmacology.

[61] Stoffel E.C., Ulibarri C.M., Folk J.E., Rice K.C., Craft R.M. (2005). Gonadal hormone modulation of mu, kappa, and delta opioid antinociception in male and female rats. J Pain.

[62] Sharp J.L., Pearson T., Smith M.A. (2022). Sex differences in opioid receptor mediated effects: role of androgens. Neurosci Biobehav Rev.

[63] Mobbs JI, Nguyen MD, Deo O, et al. Structure-guided allosteric modulation of the delta opioid receptor. Preprint. Posted online October 17, 2015. bioRxiv 682975. 10.1101/2025.10.16.682975 PMC13594213 42768001

[64] Wang Y., Luo P., Xu H. (2026). Structure-based design of an opioid receptor modulator for enhanced morphine analgesia. Sci Adv.

[65] Deo O., Pham V., Alvi S. (2025). A structure-activity relationship study of novel positive allosteric modulators for the δ-opioid receptor. ACS Chem Neurosci.

[66] Deo O., Alvi S., Pham V. (2022). The design, synthesis, and evaluation of novel 9-arylxanthenedione-based allosteric modulators for the δ-opioid receptor. J Med Chem.

